# Vapor-Liquid Equilibrium of Carbon Dioxide With Isobutane and n-Butane: Modified Leung-Griffiths Correlation and Data Evaluation

**DOI:** 10.6028/jres.095.054

**Published:** 1990

**Authors:** James C. Rainwater, Hepburn Ingham, John J. Lynch

**Affiliations:** National Institute of Standards and Technology, Boulder, CO 80303

**Keywords:** butane, carbon dioxide, critical region, data evaluation, vapor-liquid equilibrium

## Abstract

The Leung-Griffiths model as modified by Moldover and Rainwater is used to correlate high-pressure vapor-liquid equilibria of mixtures of carbon dioxide with *n*-butane and isobutane. Model correlations are compared against 10 independent experimental sources for these mixtures. Agreement is generally very good and comparable to mutual experimental discrepancies. The utility of the model as a data evaluation technique is demonstrated in that small suspect regions have been identified in certain data sets and the model predictions have been confirmed by subsequent measurements that agree with the model better than the earlier data.

## 1. Introduction

In several previous papers, the Leung-Griffiths model [1], as modified by Moldover, Rainwater, and coworkers [2–7], has been shown to be a very useful technique for correlation of vapor-liquid equilibria (VLE) of binary mixtures over an extended critical region [8–10]. It has also been shown that the model can provide a highly plausible description of a coexistence surface from limited data [11], and therefore, that it shows some promise as a “predictive” as well as a correlative technique. We describe in the present paper our experiences in correlating with the modified Leung-Griffiths model two very similar mixtures of particular interest for enhanced oil recovery, carbon dioxide + isobutane and carbon dioxide + *n* -butane. We will show that the model can, in certain instances, identify data that are suspect or incorrect.

In the development of the model, care has been taken to avoid overfitting. For any equation of state or thermodynamic model, the use of additional parameters will lead to a closer fit to benchmark quality data. However, the addition of new parameters also increases the danger of inappropriately fitting noise or error in data of lesser quality.

Elsewhere [10], we report the results of a comprehensive literature survey of binary mixture VLE over the “extended critical region,” defined as the region from the mixture critical pressure down to one-half that pressure. Our criteria for thorough measurement essentially are that at least four isopleths (loci of constant composition) or four isotherms, more or less evenly spaced between the two pure-fluid critical points, should be measured and reported. Our survey has located 129 thoroughly measured mixtures, according to these criteria, which represent a wealth of experimental data for testing the model, but still a small fraction of the total number of binary mixtures of interest. The majority of these mixtures have been measured no more than once; the multiple measurement of carbon dioxide + *n*-butane noted in this article is quite exceptional.

While the technology to perform critical-region VLE experiments has been available for nearly a century [12, 13], the experiments remain a very tedious process and resistant to automation procedures, so that, worldwide, typically an average of only three new mixtures are thoroughly measured each year [10]. Therefore, it is unrealistic to expect that benchmark quality data for most mixtures of interest will be retaken in the near future. A correlator is then faced with the problem of developing the best possible mathematical description of the coexistence surfaces of mixtures with data from many different laboratories over a long period of time, and of widely differing precision and accuracy. For such correlations, then, it would be particularly useful to have a model which succeeds in fitting accurate data but which fails to fit, and thereby identifies as suspect, data with noise or error.

In the testing of a phase equilibrium algorithm as an evaluative technique, ideally the following scenario would confirm its utility. First, the VLE surface of a particular mixture is measured. Second, the algorithm is used to correlate the data, and it is found that most of the data can be represented accurately, but in some small regions of pressure or temperature there are irreconcilable discrepancies, thus suggesting that in those regions the data are suspect. Third, a separate and independent measurement of the mixture in the suspect regions is performed, and the new data are compared against the calculations of the model. The model can then be judged successful as an evaluative technique if the new data agree better with the model predictions than with the older data.

While such a scenario is ordinarily very difficult to organize, it has taken place during our studies of carbon dioxide with isobutane and with *n*-butane. Our case histories are described in detail in sections 3 and 4. To give a brief summary, we began this study in 1985 by fitting carbon dioxide + isobutane only with the data of Besserer and Robinson [14], although their measurements did not fully define the critical locus. It was not possible to fit the dew side of their highest isotherm (394.26 K) with any set of adjustable parameters, but other dew-bubble curves were well represented. Upon subsequently comparing with the data of Weber [15], which had just then become available, we found that the correlation agreed with the dew curve at that temperature quite accurately.

For carbon dioxide + *n*-butane, at the beginning of our study the only data available to us and considered to be useful as input were those of Weber [15] and those of Olds et al. [16]. Some additional data for this mixture had been reported by Robinson and coworkers [17, 18] and by Behrens and Sandler [19], but only over a restricted temperature range. Poettmann and Katz [20] measured VLE along isopleths up to the critical locus, without densities, for mixtures of carbon dioxide with propane, *n*-butane, and *n*-pentane. We did not include these data as input to our original correlation because they have been frequently criticized in the literature [9, 21, 22].

Since our original correlation, a remarkably large amount of additional carbon dioxide + *n*-butane VLE data has become available. In fact, it appears that carbon dioxide + *n*-butane has become somewhat of a “standard mixture” within the VLE experimental community, and that the motivation for much of the recent work has been to test a new apparatus for reliability against a well characterized mixture rather than to add to the world’s VLE database. The sources for VLE data on carbon dioxide + isobutane and carbon dioxide + *n*-butane are summarized in table 1.

The original correlation represented the isotherms of Olds et al. [16] and of Weber [15] accurately except for data near the maxcondentherm point on Weber’s dew curve at 394.26 K. Shortly thereafter, data from the thesis of Pozo de Fernandez [24], subsequently published [26], became available to us. Along her isotherms nearest to 394.26 K, her data and our model agreed remarkably well. With this result as partial motivation, Niesen [28], using the apparatus of Weber upgraded to measure densities, remeasured the 394.26 K isotherm and found much closer agreement to our model predictions than to Weber’s earlier data in that region.

The Leung-Griffiths model is briefly reviewed in section 2; it has been explained in considerably more detail elsewhere [7]. We present the data and correlation for carbon dioxide + isobutane in section 3, and for carbon dioxide + *n*-butane in section 4. These sections include many graphical illustrations of the model as applied to the various data sets. The work is summarized in section 5.

## 2. The Modified Leung-Griffiths Model

A discussion of the Leung-Griffiths formalism must necessarily begin with the distinction made by Griffiths and Wheeler [29] between “field variables” and “density variables.” In a two-phase equilibrium system of liquid and vapor, field variables have equal values in the two phases; examples are the pressure *P*, temperature *T*, and chemical potentials *µ*_1_, and *µ*_2_. By contrast, density variables have different values in the liquid and the vapor; examples are the molar density *ρ* and composition *x*.

Griffiths and Wheeler have proposed that the thermodynamic description of a mixture is simpler when expressed entirely in terms of field variables. Conventional equations of state are mixed representations of two field variables, *P* and *T*, and two density variables, *ρ* and *x.*

Furthermore, the modified Leung-Griffiths model introduces functions of field variables, themselves also field variables, that represent dimension-less “distances” from one of the pure fluids and from the critical locus. These distance variables are

$$\zeta =\frac{e^{\mu_{1}/RT}}{Ke^{\mu_{2}/RT}+e^{\mu_{1}/RT}}$$ (1)

and

$$t=\frac{T-T_{c}\left(\zeta \right)}{T_{c}\left(\zeta \right)}$$ (2)

where *R* is the gas constant, *K* can be a constant or a temperature-dependent function [7], and *T*_c_(*ζ*) is the critical temperature for the given ζ value on the critical locus.

Our (arbitrary) conventions are that fluid 1 is the less volatile component, here the butane isomer, that fluid 2 is the more volatile component, here carbon dioxide, and that *x* = 1 denotes pure fluid 2. In the limit of pure fluid 1, *µ*_2_ = −∞ and vice versa, so that ζ = 0 when *x* = 1 and ζ = 1 when *x* = 0. Loci of constant ζ on the coexistence surface are given by

$$\begin{array}{c} \frac{P}{T}=\frac{P_{c}\left(\zeta \right)}{T_{c}\left(\zeta \right)}[1+C_{3}\left(\zeta \right){\left(-t\right)}^{2-\alpha }\\ +C_{4}\left(\zeta \right)t+C_{5}\left(\zeta \right)t^{2}+C_{6}\left(\zeta \right)t^{3}] \end{array}$$ (3)

and coexisting densities as functions of ζ and *t* by

$$\rho =\rho_{c}\left(\zeta \right)[1\pm C_{1}\left(\zeta \right){\left(-t\right)}^{\beta }+C_{2}\left(\zeta \right)t]$$ (4)

where plus denotes liquid and minus vapor, and *α* and *β* are the usual critical exponents. Classically, *α* = 0 and *β* = 1/2, while according to present theoretical understanding and the most accurate experimental results to date, *α* = 0.11 and *β* = 0.325 at the critical limit. We use the “effective” values *α* = 0.1 and *β*=0.355, which differ slightly from the asymptotic or “scaling-law” values but provide a better fit over an extended critical region −0.1 <*t* <0 or, equivalently, from the critical pressure down to about half that pressure.

For ζ = 1 and ζ = 0, eqs (3) and (4) become fitting equations for the vapor pressure curves and coexistence temperature-density curves of fluids 1 and 2, respectively. The parameters for the three pure fluids of this study are listed in table 2, and the functions C*_i_*(ζ) must assume these values as boundary conditions. Within the present model, for i ≥ 3 the functions C*_i_*(ζ) are simple linear interpolations between the pure-fluid values, but the ζ-dependences of *C*_1_ and *C*_2_ are characterized by adjustable parameters *C*_x_, *C*_Y_ and *C*_R_ which are different for each mixture; see reference [6], eqs (26) and (27).

The equations for the liquid and vapor compositions are rather involved; see reference [6], eqs (18) and (19). An auxiliary function $\bar{H}\left(\zeta ,t\right)$ appearing in the equations for *x* contains the additional adjustable parameters *C*_H_, *C*_z_ and ${\bar{H}}_{1}$. The mixture parameters for the systems studied here are listed in table 3. To complete the specification of the model, the critical locus must be fitted to polynomial functions; see reference [6], eqs (21) and (24). These functions include the additional parameters *T*_i_ and ${\bar{P}}_{i},$ l ≤ *i* <4, and ${\bar{\rho }}_{i},$ l ≤ *i* ≤ 3. Parameters for the critical loci of carbon dioxide + isobutane and carbon dioxide *+ n*-butane are listed in table 4.

For the correlations presented here, the mixture parameters have been adjusted by graphical methods for a best fit according to purely visual criteria. Towards the end of this project, we succeeded in developing the first formal nonlinear fitting program for the Leung-Griffiths model [10], but this approach is in a preliminary stage at present. For example, we have not yet systematically examined different choices of an “objective function” or “distance” between theory and experiment that is to be minimized, and our formal fits have depended on initial guesses made by the older visual methods. Our main objective of this paper is not to produce the absolutely optimal correlations of mixtures of carbon dioxide and the butane isomers, but rather to show the potential of the modified Leung-Griffiths model as a data evaluation technique, and for this purpose the visual fits suffice.

## 3. Carbon Dioxide + Isobutane

**Figure 1:**
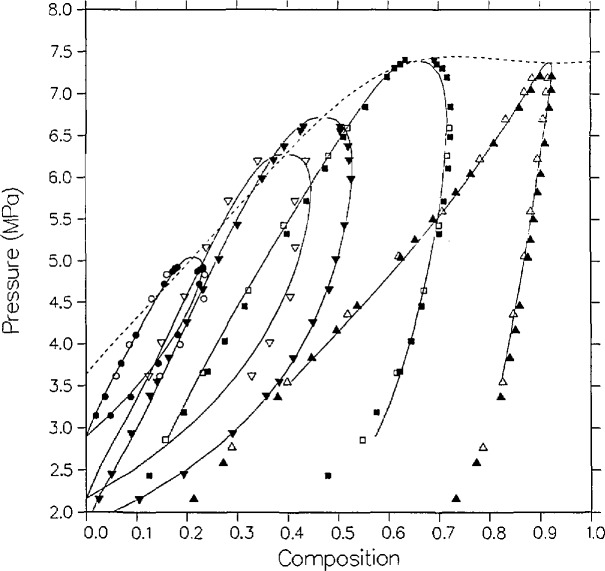
VLE isotherms for carbon dioxide + isobutane from the model as compared with the data of Besserer and Robinson [14]; ∆, 310.93 K; □, 344.26 K; ▽, 377.61 K; ◯, 394.26 K; and the data of Weber [15]; ▲, 310.93; ■, 344.26 K; ▼, 369.26 K; ●, 394.26 K. The dashed line in this and subsequent figures is the critical locus.

**Figure 2:**
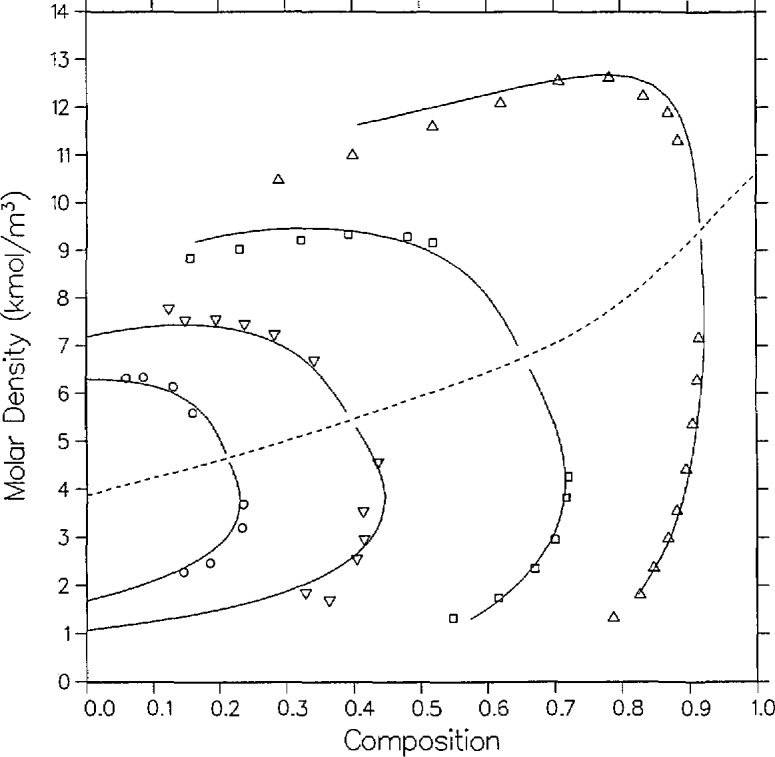
Density-composition diagram for carbon dioxide + isobutane with isotherms from the model as compared with the data of Besserer and Robinson [14]; same temperatures and symbols as figure 1.

**Figure 3:**
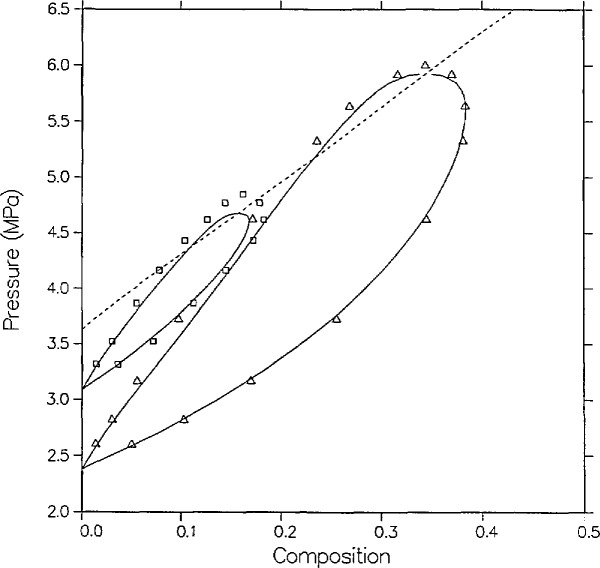
VLE isotherms for carbon dioxide + isobutane from the model as compared with the data of Leu and Robinson [25]; △, 383.15 K; □, 398.15 K.

There are three sources of experimental saturation points for the mixture carbon dioxide + isobutane. The first set was measured by Besserer and Robinson [14]. For four temperatures they provide liquid and vapor measurements of composition and density at pressures up to the critical line. Theirs are the only high-pressure coexisting density measurements available for this mixture. Weber [15], in this laboratory, has taken *P-x* data for four isotherms, three of which are at the same temperatures as those of Besserer and Robinson. Recently, Leu and Robinson [25] have published *P-x* data at two additional temperatures. These various saturation points are displayed in figures 1–3.

The parameters for the pure-fluid saturation correlations are listed in table 2. The fit for carbon dioxide is taken from Moldover and Gallagher [2] which is based on the measurements of Michels et al. [30]. That for isobutane is from the paper by Diller et al. [31] as based on the correlation of Waxman and Gallagher [32]. We first attempted a fit to the results of Besserer and Robinson. Despite the poor agreement with the dew curves of their two highest isotherms, that initial correlation agreed well with Weber’s dew curves in the same temperature range. The final correlation as presented in figures 1–3 was simultaneously optimized to both sets of data. Parameters for the critical locus are listed in table 4. For this system, six mixture parameters are necessary as listed in table 3.

The agreement between theory and experiment is best on the two lowest isotherms of Besserer and Robinson. As can be seen from figure 1, the 310.93 K isotherm of these authors is displaced by approximately 0.01 mole fraction from that of Weber, and the correlation effects a compromise between the two curves. The liquid densities of Besserer and Robinson in figure 2 are predicted to be larger than experimental values by 2 to over 5 percent on 7 of the 12 points for which *ρ* > 9.0 kmol/m^3^. A similar discrepancy is seen with carbon dioxide + *n*-butane and other similar mixtures, and is probably a minor shortcoming of the 6-parameter model.

The higher isotherms have what may be legitimately interpreted as outlier points. The vapor points at *x* =0.3288 and *x* =0.3638 on the 377.61 K isotherm are high in *x* on both the *P-x* and the *ρ*-*x* sides. It is interesting to note that Besserer and Robinson omit the *P* = 6.1984 MPa dew point in order to fit their curve. By contrast, the model indicates that the actual outlier is the next lower dew point (*P* = 5.7227 MPa) which is low in *x* by 0.03 mole fraction on both plots. On the density side, the bubble point *x* =0.1242 is high in p by about five percent, in comparison with both the model and the adjacent data.

While the scatter in vapor compositions for *T* = 377.61 K appears to be random, the dew points for *T* = 394.26 K are systematically high in composition relative to the model. The vapor points below *P* = 4.6 MPa are high in by 0.02 to 0.03 mole fraction. Note, however, from figure 1 that the model is in excellent agreement with Weber’s dew curve at the same temperature. Also, the vapor pressures measured by Besserer and Robinson at *x* = 0 (pure isobutane) at the higher temperatures are over 0.1 MPa higher than those from the correlation of Diller et al. [31].

This example shows the utility of the modified Leung-Griffiths model as both a correlative and an evaluative technique. Clearly, the model yields a highly plausible overall fit for the coexistence surface. Except for the points explicitly singled out in the above discussion, agreement is to within 0.1 MPa in pressure, 0.01 in mole fraction and 3 percent in density. Besserer and Robinson state uncertainties for their measurements but these are generally an order of magnitude lower than the discrepancies between the model and the measurements of others; especially for the higher isotherms. The underestimation of experimental uncertainty is not uncommon in VLE metrology.

This is our first case history to demonstrate that the model can lead to identification of certain data as suspect. We originally fitted the model only to the data of Besserer and Robinson, but finally had to accept the poor fit at *T* = 394.26 K. When Weber’s data subsequently became available, however, the same correlation offered excellent agreement with his dew curve at that temperature. The final correlation, of course, included Weber’s data. We also readjusted the *P-T* critical locus, since the latter’s data extends closer to the critical line. Very similar irregularities were encountered in a separate analysis of the ethane + isobutane data also measured by Besserer and Robinson [33].

The overall agreement between theory and experiment is considerably better with the data of Weber, as is illustrated in figure 1. The 344.26 K isotherm has the largest deviations. On the liquid side is a set of five points between *x* =0.2409 and *x* =0.4373 that are all low in *P* by over 0.11 MPa and high in *x* by over 0.011 mole fraction. On the vapor side, two of the points near the critical locus are high in *x* by 0.013 and 0.017 mole fraction. The *x* = 0.5760 dew point at the bottom of the extended critical region is low in *x* by 0.013 mole fraction. This may be a small defect in the model critical locus from compromises made in fitting both data sets simultaneously. Otherwise, the agreement is to within 0.08 MPa in *P* and 0.009 mole fraction in *x.*

Recently, Leu and Robinson [25] reported some new high-temperature VLE data for the same mixture. Figure 3 shows that the agreement between theory and experiment is best on the lower isotherm (383.15 K). On the higher isotherm (398.15 K) there are four points in the immediate critical region that are significantly high in *P.* Once more, though the measurements show less scatter than in the data of Besserer and Robinson [14], the *x* = 0 (isobutane) vapor pressures at which the dew and bubble curves converge are 0.08 and 0.09 MPa higher than those of the model and those from a correlation done by Diller et al. [31]. In the following section similar problems with the highest isotherms of the same authors’ recent measurements of carbon dioxide + n-butane [25] and of carbon dioxide with the pentane isomers [34,35] will be discussed.

## 4. Carbon Dioxide + *n*-Butane

The coexistence surface in the extended critical region of carbon dioxide + *n*-butane has been measured perhaps more extensively than that of any other mixture. Data have been presented by laboratories at seven different institutions: the University of Michigan [20], California Institute of Technology [16], the University of Alberta [17,18,25], the University of Delaware [19,27], Cornell University [24,26], Oklahoma State University [23], and the National Institute of Standards and Technology, Boulder [15,28]. A correlation of this mixture based on an earlier version of the model was performed with some success by Al-Sahhaf et al. [36].

This modified Leung-Griffiths correlation, presented previously by Moldover and Rainwater [6], was optimized to the data of Olds et al. [16] and Weber [15]. The parameters for pure *n*-butane in table 1 were determined by Rainwater and Williamson [5] from the data of Kay [37]. For this system, six mixture parameters (table 3) are necessary. Parameters for the critical locus are listed in table 4. Comparisons have then been made without further adjustment against the remaining data sets, many of which have been published only very recently. We present many of these comparisons graphically in this section. Some have been omitted because of space considerations.

**Figure 4:**
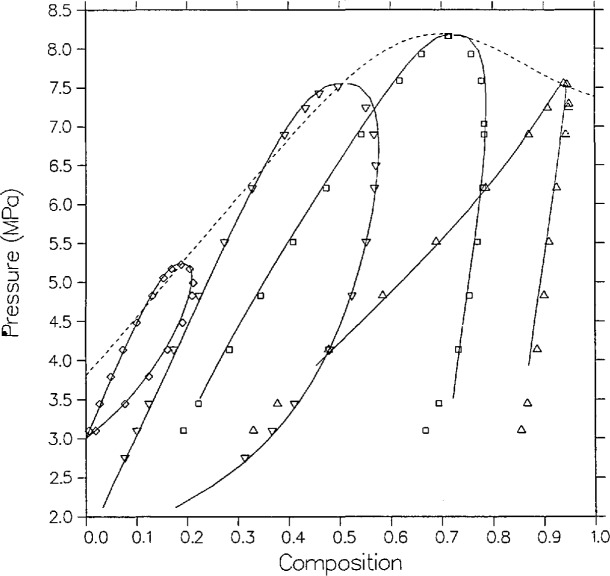
VLE isotherms for carbon dioxide + *n*-butane from the model as compared with the data of Olds et al. [16]; ∆, 310.93 K; □, 344.26 K; ▽, 377.59 K; ◊, 410.93 K.

**Figure 5:**
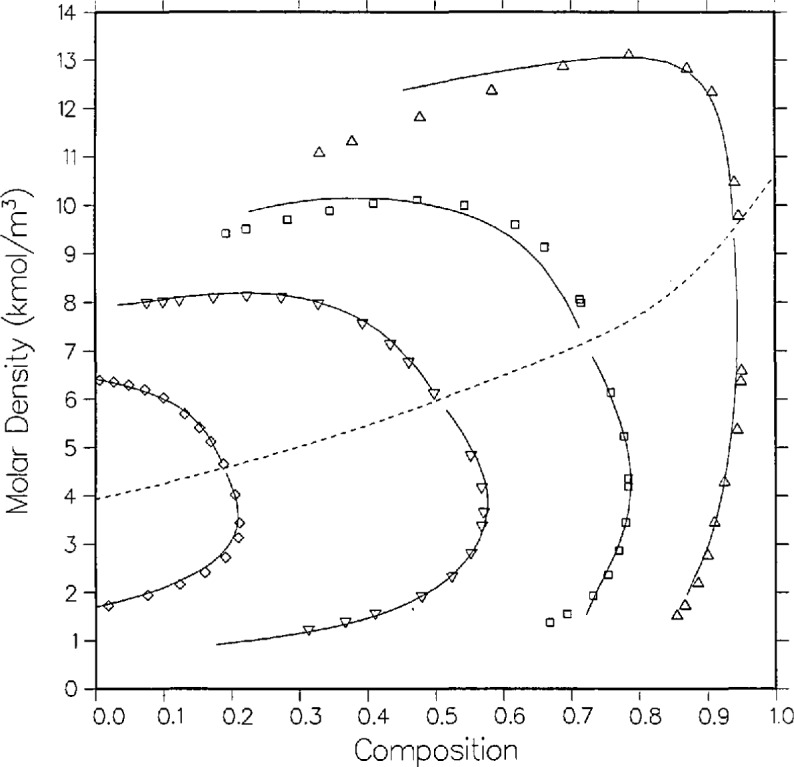
Density-composition diagram for carbon dioxide + *n*-butane with isotherms from the model as compared with the data of Olds et al. [16]; same temperatures and symbols as figure 6.

Figures 4 and 5 compare the model with the measurements of Olds et al.; their data are smoothed as explained in their article. In *P-x* space, figure 4, the agreement is quite good, within 0.01 in mole fraction except for the vapor points nearest the critical point on the 344.261 and 377.594 K isotherms. During the time of their experiments dew-bubble curves were usually assumed to have a parabolic shape. This assumption yields a critical exponent *β* of 1/2 which would greatly affect their data smoothing. Modern scaling-law theory, contained within the Leung-Griffiths model, has predicted that *β* equals approximately 1/3.

As seen from figure 5, coexisting density predictions are quite accurate on the 410.928 and 377.594 K isotherms, within 0.06 kmol/m^3^ and 0.01 mole fraction. However, as with carbon dioxide + isobutane, there are systematic deviations in the liquid densities for the two lower isotherms. Near the critical locus the densities are underpredicted by as much as 0.6 kmol/m^3^, while far from the critical locus (at *t* = −0.1, the limit of the computed curves) they are overpredicted by as much as 0.6 kmol/m^3^. Again, such discrepancies are probably due to limitations of the present model for mixtures of dissimilar fluids.

**Figure 6:**
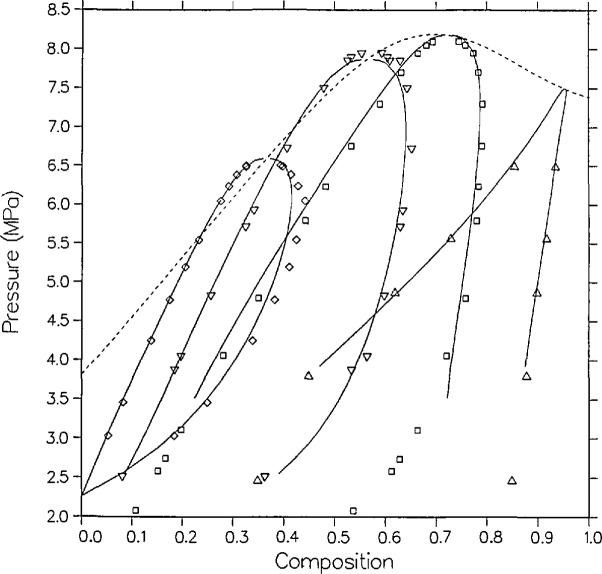
VLE isotherms for carbon dioxide + *n*-butane from the model as compared with the data of Weber [15]; ∆, 309.1 K; □, 344.26 K; ▽, 369.26 K; ◊, 394.26 K.

The model is compared with the data of Weber [15], who did not measure coexisting densities, in figure 6. Agreement with Weber’s isotherm at 309.1 K is somewhat better than with the 310.93 K isotherm of Olds et al. There are three noticeable systematic discrepancies between the correlation and Weber’s measurements, one more serious than the others.

On the bubble side at 344.26 K, the compositions as predicted by the model are lower than the data by 0.009 to 0.018 mole fraction; this is further discussed below. On the 369.26 K isotherm the results near the critical point and on the dew side for P>5.0 MPa suggest a mismatch in critical temperature between the model and Weber’s results near *x* = 0.59 to *x* = 0.65 of about 1 to 2 K. This could be remedied with a revised fit but at the expense of the good agreement between the model and the neighboring isotherm of Olds et al. The parameters of the correlation represent the best mutual optimization of Weber’s data and those of Olds et al.

**Figure 7:**
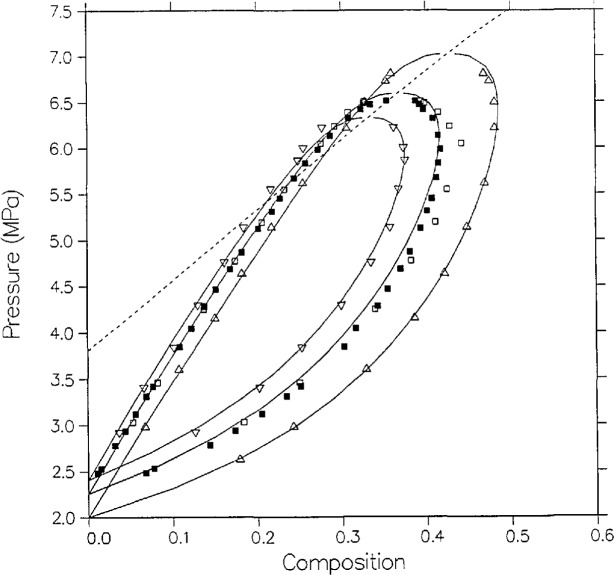
Comparison of the model predictions and various experimental isotherms for carbon dioxide + *n*-butane; ∆, Pozo de Fernandez [24], 387.62 K; □, Weber [15], 394.26 K; ■, Niesen [28], 394.26 K; ▽, Pozo de Fernandez [24], 397.89 **K.**

The most serious discrepancy is on the dew side of the 394.26 K isotherm between 4.8 and 6.2 MPa. The experimental results show a larger composition than the model predictions by as much as 0.026 mole fraction at *x* =0.442. A close fit to these vapor points was not possible. Data that became available to us subsequent to this correlation strongly support the hypothesis that the model, rather than the dew curve of Weber at 394.26 K, is correct. Figure 7 shows the model predictions, the isotherm of Weber, and a remeasurement of the same isotherm by Niesen [28] at 394.25 K as well as the isothermal data of Pozo de Fernandez [24,26] and model predictions at 387.62 and 397.89 K. Except for part of Weber’s dew curve and very near the critical point of Niesen (where there is a slight mismatch of critical pressure between the data and the model), the theoretical and experimental results at 394.26 K agree to within 0.01 mole fraction and 0.06 MPa. Most importantly, the maximum vapor composition (maxcondentherm point) of Niesen, *x* = 0.417, is in excellent agreement with the model prediction of *x* = 0.416 and differs substantially from Weber’s result of *x* =0.442.

**Figure 8:**
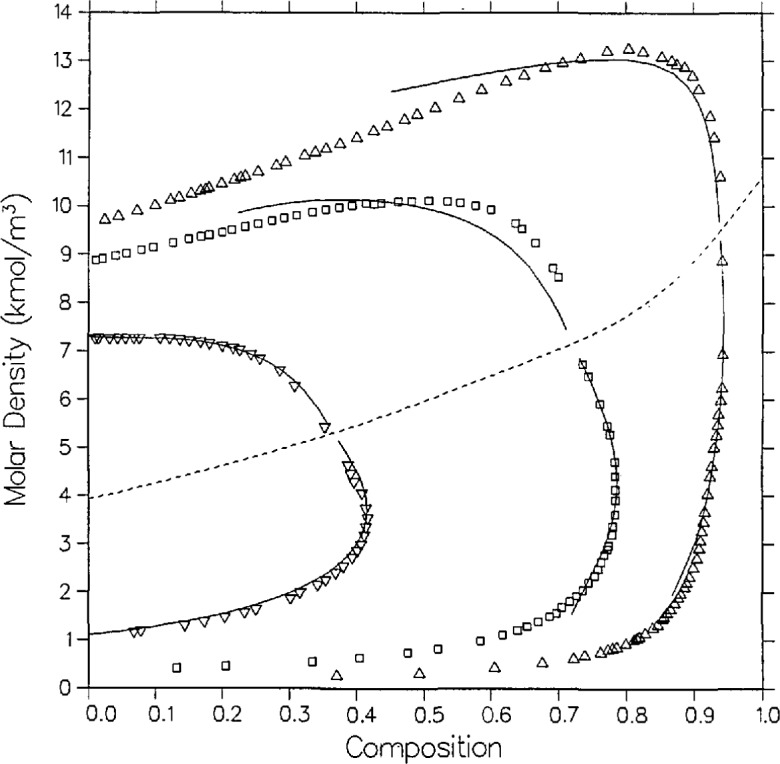
Density-composition diagram for carbon dioxide + *n*-butane with isotherms from the model as compared with the data of Niesen [28]; ∆, 311.09 K; □, 344.43 K; ▽, 394.26 K.

**Figure 9:**
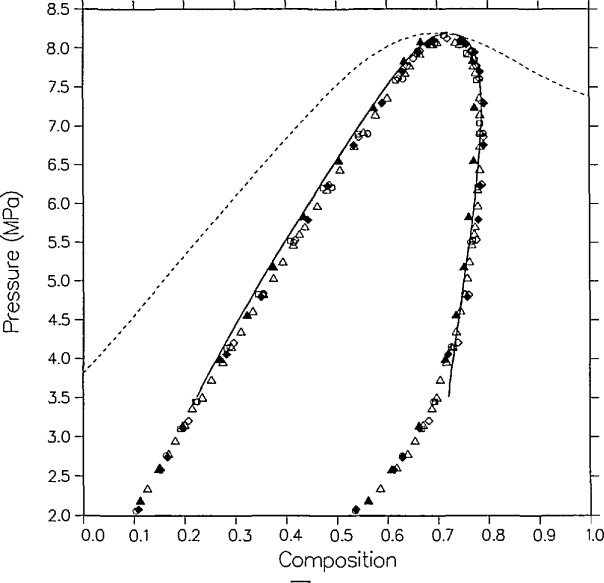
Comparison of the model prediction and five independent experimental isotherms for carbon dioxide + *n*-butane at *T* = 344.26±0.2 K (160 °F); △, Niesen [28]; □, Olds et al. [16]; ▲, Pozo de Fernandez [24]; ◊. Hsu et al. [23]; ◯, Shibata et al. [27]; ■, Weber [15].

**Figure 10:**
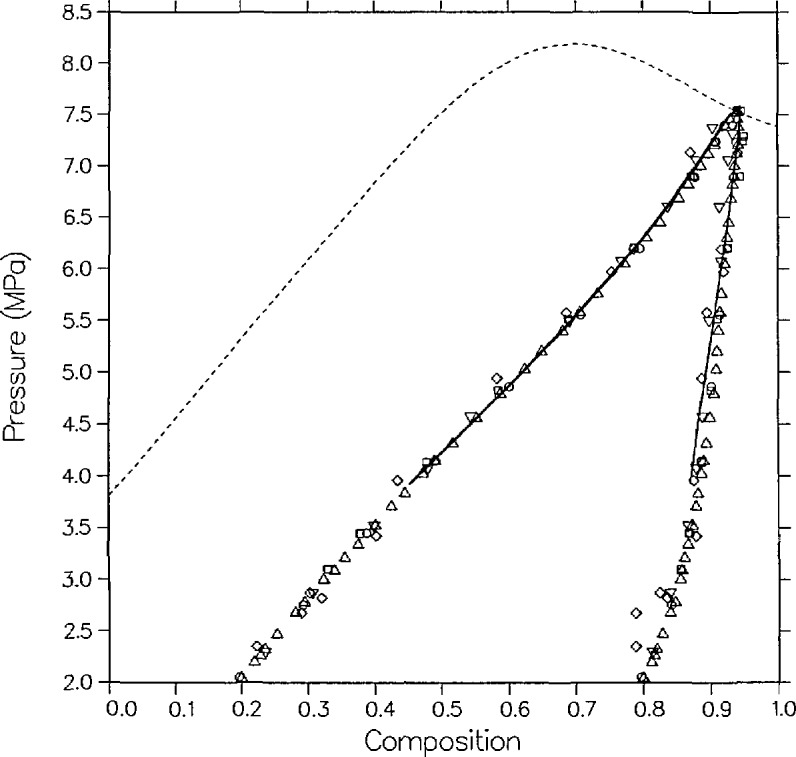
Comparison of the model predictions and four independent experimental isotherms for carbon dioxide + *n*-butane at *T*=310.9±0.3 K (100 °F); ∆, Niesen [28]; □, Olds et al. [16]; ▽, Besserer and Robinson [17]; ◊, Behrens and Sandler [19]; ◯, Shibata et al. [27].

A full comparison of the model with Niesen’s pressure measurements is illustrated by figures 7, 9, and 10. The 344.43 K isotherm in figure 9 is discussed below. Agreement with the bubble curve at *T* =311.09 K in *P-x* space is excellent, within 0.01 in composition, whereas the model systematically predicts lower vapor compositions away from the critical point by as much as 0.017 mole fraction. As with Olds et al., the densities in figure 8 are best predicted on the highest isotherm, 394.26 K, where the model agrees with experiment on the liquid side by 0.026 kmol/m^3^ and 0.007 mole fraction and on the vapor side by 0.13 kmol/m^3^ and 0.01 mole fraction. Again, as with the data of Olds et al., the discrepancies at the lower isotherms are probably due to limitations of the model.

As shown by Moldover and Rainwater [6], the agreement between the model and the data of Hsu et al. [23] is quite similar to that of Niesen. The critical pressure of the lowest isotherm of Hsu et al., however, is lower than the correlation by 0.1 MPa. Excellent agreement between the model and Niesen’s low-temperature data near the critical point lead us to conclude that the critical pressure reported by Hsu et al. is too low. It is worth mentioning, however, that Morrison and Kincaid [38] found a small minimum in critical pressure for dilute *n*-butane in carbon dioxide.

Six independent sources have reported dew and bubble points close to *T* = 344.26 K (160 °F). Figure 9 shows these results as well as the model predictions. On the liquid side, the model agrees best with the data of Olds et al. and of Pozo de Fernandez, but is systematically low in composition compared with the data of Weber, Hsu et al., Shibata, and Niesen by approximately 0.01 to 0.02 mole fraction. On the vapor side, the model shows a lack of curvature relative to the data, and predicts higher compositions than Pozo de Fernandez by more than 0.01 mole fraction but lower compositions than Hsu et al. by approximately 0.01 mole fraction.

Also, five sources have reported dew and bubble points close to *T* = 310.9 K (100 °F). Figure 10 shows these results as well as the model’s predictions. The liquid points of Behrens and Sandler are low in x by 0.02 to 0.03 mole fraction compared to the others and their vapor points show quite a bit of scatter. Near the critical locus, the isotherm of Besserer and Robinson [17] in both *P-x* space and *ρ-x* space is low in *x* by roughly 0.015 mole fraction.

Of course, it is clear from the figures that there are systematic discrepancies among the data sets by as much as 0.03 in composition. Such systematic differences among results of reputable investigators is not uncommon. In fact, disagreement among experimentalists is often an order of magnitude greater than their stated uncertainties. The modified Leung-Griffiths model can fit a particular dew-bubble curve more closely than the agreement between measurements from separate laboratories. However, in the current case it cannot be determined from the model alone which of these dew-bubble curves is correct.

Robinson and coworkers have published five isotherms (not shown) of carbon dioxide + *n*-butane over a period of years [17,18,25]. The isotherms at 283.15 K [18] and 310.85 K [17] are in fair agreement with the model, though the vapor compositions at 310.85 K appear to have some significant random scatter. These two lowest (and earliest) isotherms, unlike the other three [25], include coexisting densities. The 283.15 K isotherm is below the critical temperature of carbon dioxide and barely crosses the extended critical region −0.1< *t* <0. The model overestimates the liquid densities by 0.9 kmol/m^3^.

On the isotherms at 368.15 and 393.15 K the bubble sides agree well with the model, to within 0.009 mole fraction and 0.07 MPa. But on the dew sides, much like Weber’s questionable dew curve, the experimental compositions are higher than those of the model by as much as 0.025 mole fraction. Comparison with the model and the data sets of other experimentalists suggests that Leu and Robinson [25] were unintentionally recording vapor points at slightly lower temperatures. In fact, their data at 418.15 K agree with the model’s predictions at a temperature 1.5 K lower. The model is not likely to be in error here since it agrees almost perfectly with the isotherm of Pozo de Fernandez at the nearly identical temperature of 418.48 K. Furthermore, according to Rainwater and Moldover [4], their vapor pressure for pure *n*-butane is low and corresponds to a temperature also about 1.5 K lower. We conclude that there is a systematic experimental error in the apparatus of the University of Alberta group at high temperatures, which also affected their 398.15 K isotherm of carbon dioxide + isobutane.

**Figure 11:**
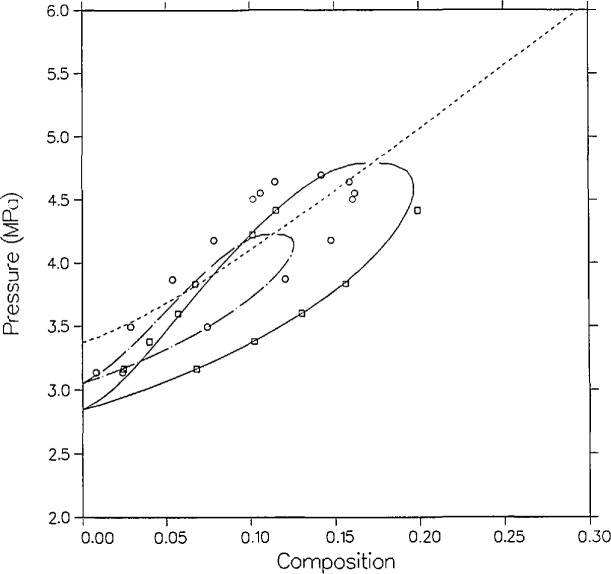
Comparison of prediction from a formal nonlinear VLE correlation of carbon dioxide + *n*-pentane with the isotherms of Cheng et al. [39]; □, 458.54 K; and of Leu and Robinson [34]; ◯, 463.15 K.

This systematic error in the measurements of Leu and Robinson can be seen especially clearly at the highest temperatures of their VLE data for mixtures of carbon dioxide with pentane isomers [34,35]. These mixtures have also been recently measured by the Cornell group; carbon dioxide with *n*-pentane [39], isopentane [40], and neopentane [41]. Though these mixtures are more difficult to correlate because the dissimilarity of the components is greater, we have developed quality correlations in *P-T-x* space for these three mixtures [10]. Figure 11 shows the correlation of carbon dioxide + *n*-pentane for pentane-rich mixtures as compared with the 458.54 K isotherm of Cheng et al. [39] and the 463.15 K isotherm of Leu and Robinson [34]. The same systematic error is evident with the other carbon dioxide + pentane isomer mixtures as with this mixture.

Behrens and Sandler [19] have reported a single isotherm of carbon dioxide + *n*-butane at 310.85 K without coexisting densities. Shibata et al. [27] remeasured this isotherm and measured two additional ones all with coexisting densities. These data are not shown, but there is much scatter in composition in the data of Behrens and Sandler, particularly at lower pressure; data of reference [27] are much more smooth. The model clearly agrees with the lone isotherm of Behrens and Sandler within the scatter, and is in good agreement with the data of Shibata et al., notwithstanding the discrepancy already noted on the 344.3 K bubble curve.

Finally, we consider the original VLE data for this mixture, the work of Poettmann and Katz [20], measured along isopleths (loci of constant composition) rather than isotherms. This work has been frequently criticized in the literature. Their critical locus for carbon dioxide + propane is drastically in error for pressure, as pointed out first by Roof and Baron [21] and more definitively by Niesen and Rainwater [9]. Despite these problems, we show here that their dew-bubble measurements of carbon dioxide + *n*-butane may have been substantially correct except for the composition measurements of their fixed samples.

**Figure 12:**
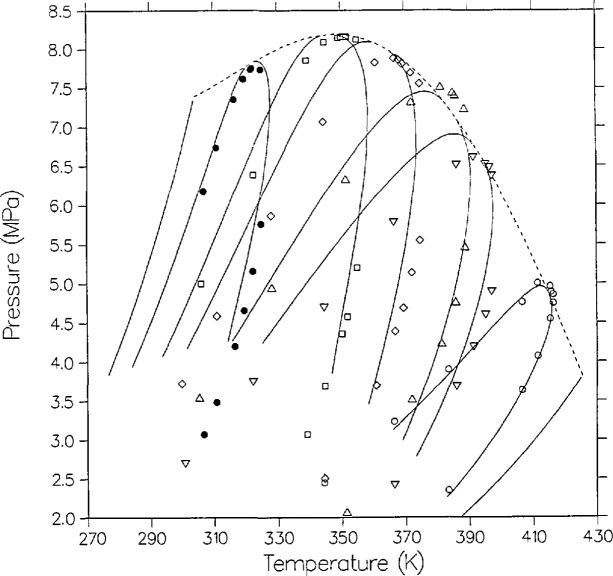
VLE isopleths (loci of constant composition) from the model as compared with the data of Poettmann and Katz [20], with the following stated compositions: ◯, 0.1393; ▽, 0.3761; ∆, 0.4551; ◊, 0.6073; □, 0.7102; ■, 0.8609.

Figure 12 shows their data in *P-T* space and the model predictions for their stated compositions (0.1393, 0.3761, 0.4551, 0.6073, 0.7102, 0.8609). Note that on a *P-T* plot, the critical locus is the envelope of the constant composition dew-bubble curves, whereas on the *P-x* plots considered earlier the critical line is the locus of maximum pressure points of isothermal dew-bubble curves. Except for the dew-bubble curve closest to pure *n*-butane, the agreement is poor.

**Figure 13:**
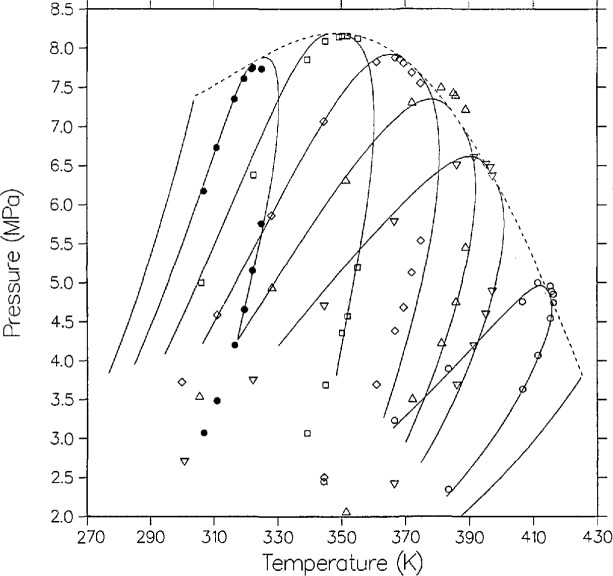
Same as figure 12 except that the model isopleths are at shifted compositions, from right to left 0.14, 0.34, 0.44, 0.55, 0.70 and 0.85.

There is evidence, however, that the University of Michigan group determined compositions inaccurately. Elsewhere [42], we have analyzed the ethylene + *n*-butane VLE data of Williams [43] from the same laboratory, and found that the dew-bubble isopleths can only be fitted if the stated compositions are shifted. Figure 13 shows the same data but with model dew-bubble curves shifted in composition (0.14, 0.34, 0.44, 0.55, 0.70, and 0.85, respectively). The agreement is much improved and the only substantial discrepancies are near the critical point of the presumed *x* = 0.44 isopleth, where the data are high in pressure by about 0.25 MPa, and on the dew side of the presumed *x* = 0.55 isopleth, where the data are low in temperature by about 4 K. Our conclusion is that Poettmann and Katz performed their experiments with reasonable accuracy in *P* and *T* for their time, before modern spectroscopic techniques had become available, but the compositions of their samples were closer to our shifted values.

## 5. Conclusions

We have analyzed in detail the binary mixtures carbon dioxide + isobutane and carbon dioxide *+ n*-butane with the Leung-Griffiths model as modified by Moldover, Rainwater, and coworkers. The model with six mixture parameters provides an excellent representation of the coexistence surface in *P-T-x* space, except perhaps for the curvature of the dew curves in the range 305 K <*T* <350 K. It does not quite reproduce properly the coexisting density curves, particularly at the lower temperatures. Our extensive work has shown that as the fluids in a binary mixture become more highly dissimilar the minor problems encountered in this work with densities become major problems but with similar kinds of disagreement between theory and experiment. The incorporation of extended scaling, as first suggested by Wegner [44], could reduce these disagreements between the model and the measurements. A generalization of the model with extended scaling is currently under investigation.

Because additional data for these mixtures, particularly the *n*-butane mixture, became available very recently and after our initial correlations, we have been able to study the merits of the modified Leung-Griffiths model as an evaluative, as well as a correlative, technique. For the isobutane mixture, the correlation was first attempted with only the data of Besserer and Robinson [14] as input but with significant discrepancies from their dew curve at 394.26 K. Weber’s measurements [15] at the same temperature then agreed with the model and the fit was revised for optimal overall agreement with both experiments. For the *n*-butane mixture, the correlation was optimized to the results of Olds et al. [16] and of Weber [15], though it was not possible to fit Weber’s dew curve at 369.26 K. Subsequent measurements by Pozo de Fernandez [24,26] and by Niesen [28] agreed much better with the model. Also the shapes of some dew-bubble curves of Olds et al. near the critical point were not quite reproduced by the model, but the predicted shapes agreed with the subsequent data of Niesen and of Hsu et al. [23]. Once again the high-temperature data of Leu and Robinson [25] did not agree with the optimized fit. We conclude that in many instances, if the model fails to correlate a certain limited feature of otherwise consistent data, the model may well be more reliable. However, when comparing different experiments there can also be small systematic errors among which the modified Leung-Griffiths formalism cannot discriminate, as shown by figures 9 and 10.

## Figures and Tables

**Table 1:** Data sources

Experimentalists		Isotherms (K)
	CO_2_+*i*-Butane	
Besserer and Robinson [14]		310.93, 344.26, 377.61, 394.26
Leu and Robinson [25]		383.15, 398.15
Weber [15]		310.93, 344.26, 369.26, 394.26
	CO_2_+*n*-Butane	
Niesen [28]		311.09, 344.43, 394.26
Olds et al. [16]		310.928, 344.261, 377.594, 410.928
Pozo de Fernandez et al. [24, 26]		292.6, 325.01, 344.25, 357.77, 377.55, 387.62, 397.89, 418.48
Besserer and Robinson [17]		310.85
Kalra et al. [18]		283.15
Leu and Robinson [25]		368.15, 393.15,418.15
Hsu et al. [23]		319.3, 344.3, 377.6
Behrens and Sandler [19]		310.85
Shibata et al. [27]		310.9, 344.3, 410.9
Weber [15]		309.1, 344.26, 369.26, 394.26
	CO_2_+*n*-Butane	
Poettmann and Katz [20]		0.1393, 0.3761, 0.4551, 0.6073, 0.7102, 0.8609 (mole fraction CO_2_)

**Table 2:** Pure-fluid parameters

	CO_2_	*iC*_4_	*nC*_4_
*T*_c_(K)	304.17	407.84	425.38
*ρ*_c_ (kmol/m^3^)	10.620	3.880	3.936
*ρ*_c_ (MPa)	7.386	3.629	3.809
*C*_1_	2.009	1.9843	1.991
*C*_2_	−0.995	−0.8738	−0.912
*C*_3_	30.870	30.223	30.000
*C*_4_	5.997	5.8742	5.99
*C*_5_	−26.130	−25.4473	−24.42
*C*_5_	−5.490	−3.2867	0.0

**Table 3:** Mixture parameters

	CO_2_-*iC*_4_	CO_2_-*nC*_4_
α_2m_	0.272	0.304
*C*_H_	−10	−14
*C*_x_	0.9	0.9
*C*_z_	−0.5	−1.0
*C*_R_	4.0	4.5
*C*_Y_	−0.2	−0.2
*H*_1_	−0.2	−0.2

**Table 4:** Critical-line parameters

	CO_2_-*iC*_4_	CO_2_-*nC*_4_
*T*_1_ [(kmol/m^3^)/MPa]	−0.063764	−0.084643
*T*_2_ [(kmol/m^3^)/MPa]	−0.029541	0.000818
*T*_3_ [(kmol/m^3^)/MPa]	0.045235	0.045271
*T*_4_ [(kmol/m^3^)/MPa]	−0.006689	−0.030294
*P*_1_ (kmol/m^3^)	2.1383	3.0729
*P*_2_ (kmol/m^3^)	1.1390	1.1274
*P*_3_ (kmol/m^3^)	−0.0228	−0.4107
*P*_4_ (kmol/m^3^)	0.1010	−0.1657
${\bar{\rho }}_{1}$ (kmol/m^3^)	−2.6851	−1.8705
${\bar{\rho }}_{2}$ (kmol/m^3^)	5.4864	5.3753
${\bar{\rho }}_{3}$ (kmol/m^3^)	−0.9751	−2.9065
